# Real-Time Tracking of COVID-19 Rumors Using Community-Based Methods in Côte d'Ivoire

**DOI:** 10.9745/GHSP-D-21-00031

**Published:** 2021-06-30

**Authors:** Natalie Tibbels, Abdul Dosso, Aliya Allen-Valley, William Benie, Corinne Fordham, Jeanne Aka Brou, Marjorie Nana, Valère Zounneme, Korodénin Fatoumata Silué, Diarra Kamara, Danielle Naugle

**Affiliations:** a Johns Hopkins Bloomberg School of Public Health, Center for Communication Programs, Baltimore, Maryland, USA.; b Johns Hopkins Center for Communication Programs-Côte d'Ivoire, Abidjan, Côte d'Ivoire.

## Abstract

Global misinformation and information overload have characterized the coronavirus disease (COVID-19) pandemic. Rumors are unverified pieces of information spreading online or person-to-person that reduce trust in health authorities and create barriers to protective practices. Risk communication and community engagement can increase transparency, build trust, and stop the spread of rumors. Building on previous work on Ebola and Zika viruses using Global Health Security Agenda systems strengthening support, the U.S. Agency for International Development-funded Breakthrough ACTION project developed a process and technology for systematically collecting, analyzing, and addressing COVID-19 rumors in real-time in Côte d'Ivoire. Rumors were submitted through community-based contributors and collected from callers to the national hotlines and then processed on a cloud-hosted database built on the open-source software District Health Information System 2 (DHIS2). Hotline teleoperators and data managers coded rumors in near-real-time according to behavioral theory frameworks within DHIS2 and visualized the findings on custom dashboards. The analysis and response were done in full collaboration with the Government of Côte d'Ivoire and implementing partners to ensure a timely and coordinated response. The system captured both widespread rumors consistent with misinformation in other settings, such as suspicions about case counts and the belief that masks were deliberately contaminated, as well as very localized beliefs related to specific influencers. The qualitative findings provided rapid insights on circulating beliefs, enabling risk communicators to nuance and tailor messaging around COVID-19.

## Abstract (precis)

Addressing rumors is critical for managing and ending a public health emergency. We piloted a system for real-time rumor tracking using community-based collection methods, open-source software, and a rapid coding and visualization process to systematically understand and help actors respond to COVID-19 misinformation in Côte d'Ivoire.

## Teaser

**Key Findings:**

- Rumors and misinformation undermine public health by creating barriers to protective practices and reducing trust in health responders.
- We developed an approach to real-time rumor tracking that leverages existing structures (like national hotlines) and local liaisons (such as community health workers) to submit rumors and misinformation to a central database. The rumors are rapidly coded using a standard form and visualized on custom dashboards.
- During the 6-month pilot of this tool in Côte d'Ivoire, we received and coded 1,757 submissions and used the thematic findings to inform risk communication and community engagement activities.

**Key Implications:**

- Health leaders should consider using a real-time rumor-tracking approach during public health emergencies and can build on the detailed guide and District Health Information System 2 metadata package provided for a simple, open-source, community-centered technology to make rumor data digestible to responders.

Resumen en français à la fin de l'article.

## INTRODUCTION

Rumors are “collective hypotheses” about reality that are communicated within groups of people, and their defining characteristic is that they are unverified or lack evidence.^1^ Rumor content has been classified based on the concerns of the group, such as speculations about causes of illness or safety of treatments. Alternatively, rumors may be based on the motivations of those spreading the rumor, such as to hurt or to help others or to express one's dread or wishes.^1^^,^^2^ Specifically, rumors containing inaccurate information can be spread with the intention of helping others or making sense of difficult experiences (misinformation), or rumors can be propagated intentionally to cause harm or confusion to others (disinformation).^2^^,^^3^ While rumors are often seen to be innocuous, those that contain harmful information can cause both morbidity and mortality and extend the life of a public health emergency. For example, in early 2020, a rumor circulating in Iran—that consuming alcohol (including disinfectants) could prevent or treat severe acute respiratory syndrome coronavirus 2 infection—coincided with 800 deaths from methanol poisoning.^4^ Influxes in the spread of rumors are often observed during epidemics, such as the current coronavirus disease (COVID-19) crisis, which has more recently been defined as an “infodemic” by the World Health Organization.^5^ The term infodemic describes a situation where there is too much information circulating about a given topic and the information contains both accurate and inaccurate statements that make it difficult for people to distinguish one from the other.^6^

During the current COVID-19 pandemic, misinformation has spread widely throughout communities and social networks alike.^6^^,^^7^ Rumors about this novel pathogen and its associated illness, COVID-19, have flourished, complicating individuals' ability to determine which information about the virus can and should be trusted. Rumors are notoriously difficult to define, identify, and track.^6^ Researchers have characterized the spread of rumors using an epidemiological framework, even estimating the “infectiousness” or pace of sharing particular pieces of information and looking at mediating factors such as linguistic features, message believability, individual personality, or group trust.^8^^–^^10^ While, on the one hand, researchers have quantified rumor dynamics, public health professionals and policy makers have oversimplified rumors as simply myths or folklore to be corrected.^11^ Stories characterized as “rumors” (such as rumors of abuse by humanitarian aid workers or secret testing of medical treatments using coercion or without consent) may later prove true and may have far-reaching impacts.^12^ Even regarding rumors that are “false,” characterizing rumors in a fact-or-falsehood dichotomy runs the risk of bringing an overly biomedical or colonial framing, thereby missing how communities conceptualize their experiences and likewise missing important insights for the design of effective prevention and control efforts.^11^

Nevertheless, developing methods to track, prioritize, and respond to harmful rumors is important to the implementation of social and behavior change interventions during public health emergencies. As a result of the uptick in social media usage in modern times, researchers are conducting studies on the diffusion of misinformation through social media platforms.^10^^,^^13^ However, while social media-based analyses provide access to large amounts of information in short amounts of time, they are limited by their inability to include the perspectives of those who do not have access to or do not use social media. Globally, social media penetration has grown to 53%, but in many settings, fewer than a quarter of the population actively access social media.^14^ Additionally, rumors may spread rapidly outside social media as individuals share information in common physical spaces or private short message service (SMS) chats that are inaccessible by social media monitoring methods. Systematically identifying and tracking misinformation that flows through “unplugged” homes and communities is necessary to a robust public health response. Surveys may identify these beliefs at certain time points, but household-based and mobile surveys typically occur infrequently and can be resource-intensive, while rumors evolve constantly and require a more agile approach.

Developing methods to track, prioritize, and respond to harmful rumors is important to implementing social and behavior change interventions during public health emergencies.

The solution to this development challenge may lie in a long-tested method recently given new life through mobile technology: real-time monitoring. In the development and humanitarian aid world, monitoring is the process of simple yet continuous data collection using standard tools to track clearly defined metrics. Mobile devices have allowed monitoring to become more rapid, with data transferred instantly to a central database for processing and analysis and visuals automatically populated to reflect realities hundreds or thousands of miles away. The application to rumor management is clear. Local cadres such as community health workers, hotline teleoperators, or health promoters may be employed as contributors who document and submit rumors they hear during their work and daily lives. Real-time rumor tracking is a relatively novel methodology, versions of which have been implemented by organizations like Internews, International Federation of Red Cross and Red Crescent Societies, United Nations Children's Fund, and Johns Hopkins Center for Communication Programs (CCP) during outbreaks of Ebola or other humanitarian emergencies.^2^^,^^15^^–^^17^ In some cases, the approach functioned like a general community feedback system, where individuals in a refugee camp, for example, could submit rumors or complaints that would be followed up by staff. In other cases, as with the DeySey system in Liberia during the Ebola outbreak of 2014–2016, the system tracked rumors specifically related to Ebola using an SMS system.^16^

CCP, with funding from the U.S. Agency for International Development (USAID) under the flagship social and behavior change program Breakthrough ACTION, piloted a real-time rumor-tracking system in Côte d'Ivoire to identify, rapidly analyze, and respond to rumors that emerged around COVID-19. Initially developed as part of the Global Health Security Agenda portfolio to address rumors related to the country's 5 priority zoonotic disease groups, the launch of the system on March 1, 2020, coincided with the first case of COVID-19 in Côte d'Ivoire on March 11. In the following sections, we summarize the multipronged community-based approach to rumor collection, the process of coding and managing rumor submissions, and summarize user feedback and lessons learned for tracking rumors during a public health emergency.

## METHODS

### Training

The 6-month pilot took place between March 1 and August 31, 2020. In February 2020, the team recruited and trained community contributors (CCs), individuals who were in touch with communities and working in fields relevant to human or animal health. A total of 20 CCs were trained: 12 in Abidjan, the capital of Côte d'Ivoire, and 8 in Bouaké, the second-largest city. They received a small amount of mobile airtime credit to allow them to submit rumors through WhatsApp. They were not required to be professional health communicators or health workers, but they were sufficiently able to recognize reportable rumors and submit them via written text or voice message. Teleoperators at 3 national health-related hotlines were also trained, including the 143 line, which led the implementation during the pilot period. The 143 line has national coverage, is free of charge to callers, and offers information and responds to the concerns of the population related to health services. The line is managed by the Government of Côte d'Ivoire and is affiliated with the Department of Communication and Public Relations within the Ministry of Health.

The training was a 2-day workshop explaining the purpose and public health benefits of tracking rumors. Participants watched videos, worked on identifying and classifying rumors, and learned how to submit rumors through WhatsApp or the digital application. CCs and teleoperators practiced recognizing reportable rumors related to any of the country's priority zoonotic diseases including COVID-19. During the training and through prompts, we characterized a rumor as a piece of information that they were not sure was true. The data management team considered rumors as either unable to be fully verified (for example, theories about where the virus originated) or verified to be false (for example, that COVID-19 cannot affect Africans). However, while the team invested considerable time during and after the training to arrive at a clear definition of a rumor for the CCs and teleoperators, we avoided asking them to be scientific experts and encouraged them to err on the side of inclusion if unsure.

### Rumor Submission

When they heard or saw something they believed to be a rumor, CCs submitted it verbatim, to the extent possible, using a text or voice message to a dedicated WhatsApp line. Message threads were individual as opposed to group chat conversations to avoid amplifying misinformation among key informants. Likewise, teleoperators who heard reportable rumors during their phone calls noted them in a standard log. Rumors identified through the hotline could come from anywhere in the country, while the CCs were located in Abidjan or Bouaké. Both CCs and teleoperators were instructed to deidentify rumors before submitting them. They were also instructed to submit a rumor every time they heard it, even if they had submitted it before.

### Rumor Documentation and Topical Coding

Teleoperators from the national hotline entered rumors received through the WhatsApp line or the hotline into a predeveloped form hosted in a cloud database built on the District Health Information System 2 (DHIS2) platform. The rumor log was an event program in DHIS2, and each rumor was labeled by date, district, and the source from which it was received (either via community contributor or through the hotline). Rumors were then coded in the same form using a standard codebook for a set of defined topics such as “prevention,” “modes of transmission,” “government response,” or “estimates of case counts.” Codes were not mutually exclusive, and rumors could have multiple codes applied. For example, the “conspiracy theory” code was defined as “a theory that rejects official accounts of an event or situation in favor of secret organizations or secret plots” and could be co-applied with “treatment” or “vaccination” or other topics. The majority of codes were defined in advance based on prior research conducted on priority zoonotic diseases or based on behavioral theory such as the Extended Parallel Processing Model.^18^ This model posits that in the context of a threat, perceived susceptibility to an illness, anticipated severity of the illness (if contracted), and perceived efficacy of protective behaviors to alleviate the threat can all influence whether people feel motivated to practice protective behaviors. Rumors related to this model—including who is at risk for COVID-19, how serious COVID-19 is, and whether the recommended response measures work—were all of interest to prioritize messaging that would prompt people to take action (danger control) rather than minimize or ignore the threat (fear control). If rumors did not fall into one of the predefined topical codes, the data managers would select “other,” and if they felt that a new code may be warranted, there was an open-ended field to propose new codes. As additional codes were included to reflect new rumors, prior rumors were back coded with the new codes. Teleoperators could also flag a submission for immediate follow-up if they felt a rumor should be referred to a different authority such as the police; this was typically used for submissions about financial scams related to the pandemic or threats of violence. In those cases, the rumors were passed on to project staff who reviewed them immediately and then passed the information on to the appropriate authorities where relevant.

### Real-Time Analysis and Synthesis

CCP data managers reviewed the rumors and verified 100% of the coding. In addition, every 3 days, data managers extracted coded rumors and facilitated a rapid analysis process to identify insights and synthesize submissions into one of, ultimately, 20 belief statements. For example, a topical code might be “modes of transmission,” but the belief statement may be “COVID is spreading through contaminated masks.” The rapid analysis involved reading the coded rumor submissions, identifying the belief statements, and discussing their priority based on a set of 5 questions: (1) Has this rumor been fact-checked and found to be false? (2) If believed, could this rumor cause physical harm to people? (3) If believed, could this rumor create an obstacle to accessing health services? (4) If believed, could this rumor reduce trust in health workers or public health responders? (5) If believed, could this rumor stigmatize people perceived to be at risk or infected? Through discussion of these questions, belief statements that were deemed to warrant a potential response were added to the rumor log and back coded up to 1 month.

### Data Visualization and Sharing

Custom dashboards within DHIS2 summarized the data by district, topic, belief statement, and week, allowing for rapid analysis across multiple dimensions. Insights, including any geographic or time variation in rumors, were shared with the national Risk Communication Technical Working Group (RCTWG) during weekly coordination meetings. The RCTWG, which existed and met before COVID-19, comprises representatives from government entities in the human health, animal health, and environment sectors. This group remains a permanent mechanism for coordination around risk communication for the Government of Côte d'Ivoire. The RCTWG coordinated various public health and implementing partners to address rumors as needed. At the end of the pilot period, stakeholders provided feedback on the system to assess the usefulness and relevance of the system and to improve implementation.

The real-time rumor-tracking pilot was reviewed and approved by the Johns Hopkins University School of Public Health Institutional Review Board and the Ivoirian Comité National d'Éthique des Sciences de la Vie et de la Santé (National Ethics Committee for Life Sciences and Health in Côte d'Ivoire).

## FINDINGS

During the 6-month pilot, CCs and hotline teleoperators contributed 1,757 individual submissions to the rumor-tracking system, with CCs contributing 70% of rumors received during this period. Of the rumors where a specific illness was mentioned, 97% related to COVID-19. The Figure shows the number of submissions by topic codes.

**FIGURE:**
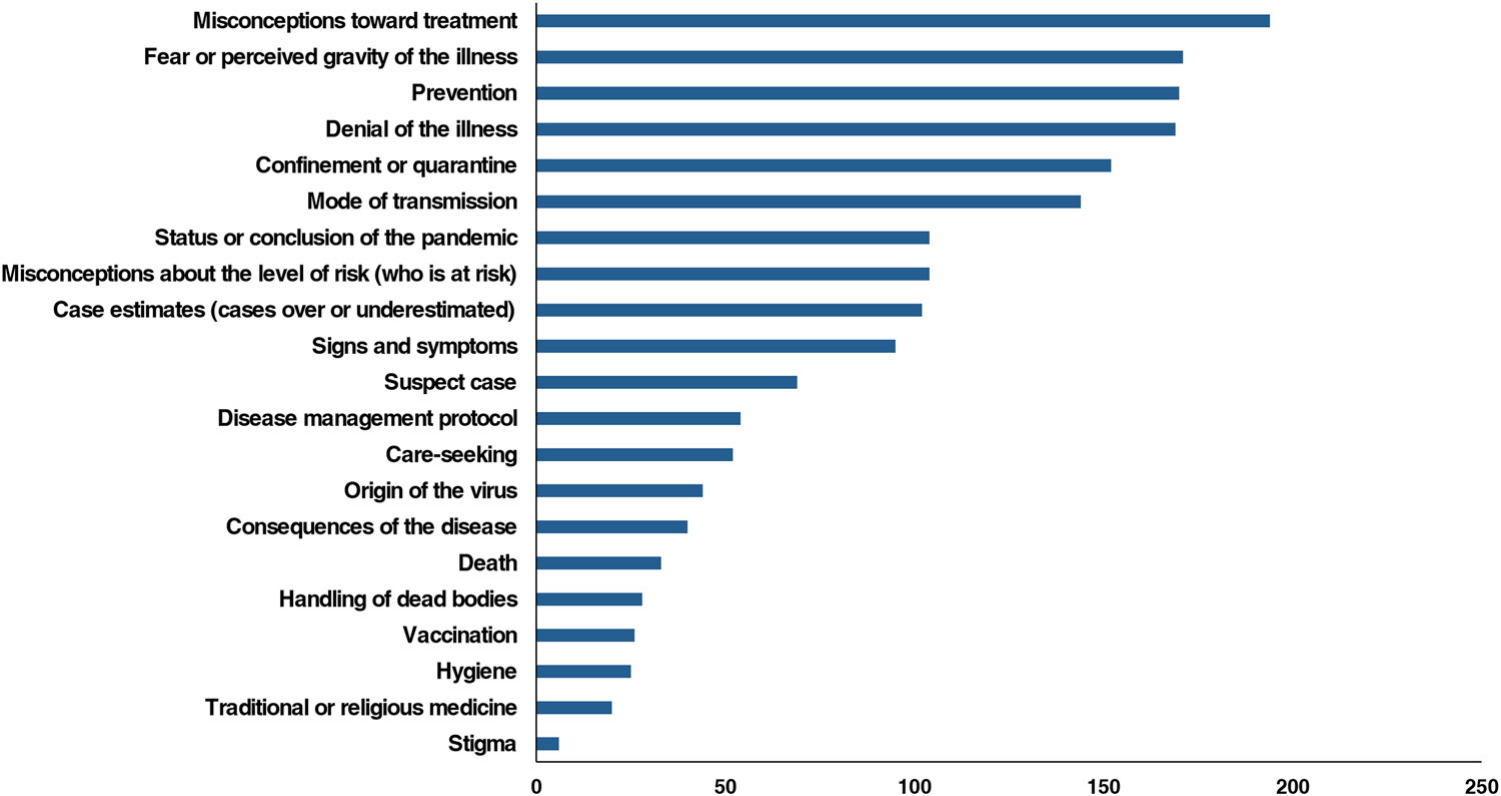
Rumors on Coronavirus Disease in Côte d'Ivoire Coded by Topic,^a^ March to August 2020 ^a^ Multiple codes could be applied to each rumor. Figure shows a subset of codes. The complete codebook can be found in the Supplement.

The most common topics related to alternative prevention and treatment methods including home and herbal remedies, perceived severity of the illness, perceptions of public health recommendations and government actions, and denial of the existence of the virus. The belief that COVID-19 does not exist or is no longer circulating in Côte d'Ivoire was consistent throughout the pilot period and frequently co-occurred with the “conspiracy theory” code. Many rumors described alternative prevention or treatment methods for COVID-19, such as consuming certain foods or drinks or medications for other illnesses (such as antiretrovirals). Communities expressed skepticism or concern about the efficacy or cost of public health recommendations or practices like wearing masks or avoiding travel. Rumors about the status and evolution of the pandemic often featured heavily, and one of the most consistent rumors was that “coronavirus is over.” Rumors related to the development of a vaccine occurred early on, then waned, and then picked up in frequency toward the end of the pilot period in August 2020.

The most common rumor topics were alternative prevention and treatment methods, perceived severity of COVID-19, perceptions of public health recommendations and government actions, and denial of the existence of COVID-19.

To further synthesize the data, belief statements were collapsed into thematic groups to organize the findings for the RCTWG. The bulk of submitted rumors fell into 1 of the following overarching themes: (1) denial of the reality of the virus or case estimates, (2) status and evolution of the pandemic, (3) recommended public health measures, (4) vaccine development and use, (5) alternative prevention and treatment methods, (6) perceived risk or susceptibility, and (7) perceived severity and symptoms. A definition of each theme is provided in Table 1.

**TABLE 1:** Overarching Themes of Rumors About COVID-19 in Côte d'Ivoire, March to August 2020

Theme	Definition
Denial of the reality of the virus and case estimates	Questions or suspicions that the virus is a complete fabrication, or that it exists elsewhere but not in Côte d'Ivoire. Beliefs that the official case counts are false or deliberately misleading (often tied to the belief that the virus is not real) for a political or financial agenda.
Origin and evolution of the pandemic	Beliefs or conspiracy theories about where the virus originated and how and why the pandemic is progressing the way it is, including the belief that the pandemic has ended.
Recommended public health measures	Beliefs about prevention measures such as masks, distancing, reducing travel, quarantining if exposed, testing, and seeking treatment. These beliefs intersect with response efficacy (whether prevention measures work to prevent COVID-19), self-efficacy (whether people feel they can practice the behaviors or what factors stop them from being able to practice the behaviors), and conspiracy theories about secret or nefarious reasons these particular measures are recommended.
Vaccine development and use	Questions or statements about the COVID-19 vaccines, including their development, authorization, testing, and implementation.
Alternative prevention and treatment methods	Suggestions or beliefs about how COVID-19 may be prevented or treated outside of the official recommendations, including home remedies and religious or traditional approaches.
Perceived risk or susceptibility	Beliefs about who is at risk of infection, severe illness, or death - including comparison with other diseases and differentiating the level of risk by various sociodemographic factors (e.g., ethnicity, climate, age, profession, and wealth).
Perceived severity and symptoms of the illness	Beliefs about whether COVID-19 is serious or not, comparisons with the seriousness of other illnesses, and beliefs about specific symptoms.

**Abbreviation**: COVID-19, coronavirus disease.

Table 2 provides a summary of the themes, belief statements, and illustrative rumors. Though each belief statement was primarily associated with 1 theme, the themes did intersect. For example, the first 2 overarching themes in Table 2 tended to overlap, with conspiracy theories about the origin and purpose of the virus (created in a lab, created to kill people) co-occurring with suspicions around the government's case and death counts being over- or underestimated for nefarious reasons. There were similar intersections between the origin theories and the perceptions of recommended health measures, specifically linking fear of testing or care-seeking to the suspicion that people were being deliberately misdiagnosed for money. Themes that were directly informed by the Extended Parallel Processing Model—such as perceptions around the personal risk of contracting the virus or the severity of COVID-19—provided rich data throughout the pilot as well as theory-informed strategies to respond to rumors through risk communication and community engagement.

**TABLE 2:** Themes, Belief Statements, and Illustrative Rumors About COVID-19 in Côte d'Ivoire, March to August 2020

Overarching Theme	Associated Belief Statements	Illustrative Rumors
Denial of the reality of the virus and case estimates	COVID-19 does not exist. The government is overestimating case numbers for financial gain. The government is hiding cases for political reasons.	*The number of people infected by coronavirus is purely a political calculation.* —CC, Bouaké, April 9, 2020 *They say that the government is increasing the number of confirmed cases of COVID-19 in order to benefit from the money that the WHO is dispersing for the countries that have coronavirus.* —CC, Abidjan, April 13, 2020 *Coronavirus never existed in Côte d'Ivoire* —CC, Bouaké, August 28, 2020
Origin and evolution of the pandemic	COVID-19 was created in a laboratory. COVID-19 is being intentionally spread to kill people. COVID-19 is over.	*Coronavirus is made up of a group of viruses that were created in a laboratory* —CC, Bouaké, June 25, 2020 *COVID-19 was created intentionally for harm.* —CC, Bouaké, July 13, 2020 *Currently, the danger is not present anymore, and they continue to instill an irrational fear.* —CC, Bouaké, August 28, 2020
Recommended public health measures	Recommended measures do not work or are dangerous. It is dangerous or useless to seek testing or treatment for COVID-19.	*Masks are infected with COVID-19. Use the ones that are created locally.* —CC, Abidjan, April 14, 2020 *They say that the COVID-19 testing lefts are the places that transmit the illness* —Hotline caller, Koumassi Port-Bouet Vridi, May 5, 2020
Vaccine development and use	A dangerous COVID-19 vaccine is disguised as a routine vaccine. A COVID-19 vaccine is designed to infect or kill the population. The state will force us to get vaccinated.	*There's a vaccine that transmits COVID-19, when you want to kill yourself, they inject you with the virus, you suffer, and after that you die.* —CC, Bouaké, July 5, 2020
Alternative prevention and treatment methods	Certain food or drinks will prevent or cure COVID-19. Washing in a certain way prevents or cures COVID-19. Certain medications prevent or treat COVID-19. Sex cures COVID-19. There is a new cure that is hidden from the population.	*Have sex constantly to kill coronavirus.* —CC, Bouaké, March 16, 2020 *It is said that the government says to eat everything that is hot and wash with hot water because it cures coronavirus.* —Hotline caller, Grand-Lahou, April 29, 2020 *They say that in Italy, the remedy against coronavirus has finally been found.* —CC, Abidjan, May 22, 2020 *Drinking cucumber water every morning is very effective for eliminating the COVID-19 virus.* —CC, Bouaké, July 7, 2020 *The medicine (remdesivir) existed before corona, they created their virus and also the medicine all this for money earned through the death of thousands of people. The world is fucked up*. —CC, Bouaké, July 7, 2020
Perceived risk or susceptibility	COVID-19 does not kill or affect certain people (e.g., age, blood type, economic status). COVID-19 does not kill or affect Africans or Black people.	*The coronavirus cannot catch a Black African because of his Black skin.* —CC, Abidjan, March 9, 2020 *The genetic composition of African blood resists coronavirus.* —CC, Bouaké, March 12, 2020 *Coronavirus doesn't kill children, but the virus attacks people who are very old and kills them.* —CC, Bouaké, July 7, 2020
Perceived severity and symptoms of the illness	Closures and the economic effects of the pandemic are worse than the virus itself. COVID-19 has weakened or is no longer a threat.	*Coronavirus kills faster than Ebola.* —Hotline caller, Yopougon-Ouest-Songon District, May 4, 2020 *Malaria is so much worse than COVID-19 in terms of mortality.* —CC, Bouaké, July 5, 2020 *Patients that survive COVID-19 always suffer from cardiac problems.* —CC, Bouaké, July 5, 2020 *One patient out of 20 will feel the symptoms of the illness again one month, even nearly 3 months after the first appearance of symptoms.* —CC, Bouaké, July 7, 2020

**Abbreviation**: CC, community contributor; COVID-19, coronavirus disease.

In response to the rumors collected via the rumor tracker, during its routine meetings, the RCTWG coordinated the risk communication and community engagement efforts alongside representatives of the Government of Côte d'Ivoire implicated in risk communication and pandemic response, such as the Center for Emergency Public Health, the Crisis Communication Committee, and implementing partners working on social and behavior change activities. The RCTWG prioritized rumors that were timely, reported multiple times, had the potential to cause harm, or put people at risk. Strategic communication messages developed based on rumor data focused on the correct information and avoided restating the rumors. Messages were disseminated through communication products such as radio spots, posters, and through influencers such as religious leaders. Under the guidance of the RCTWG and using the rumor data, the project developed and disseminated radio spots on radio stations that report a reach of 3.6 million people, as well as placing posters and billboards in neighborhoods with populations totaling over 800,000 people. As a result of themes identified in the rumor tracker highlighting the widespread skepticism about the existence of COVID-19 and low-risk perception, specific messages were added to emphasize that COVID-19 exists and is a threat to everyone. Other radio spots modeled a safe visit to a health center in response to rumors that suggested people were afraid to seek routine services. The project used its Facebook page and the public Facebook pages of the Government of Côte d'Ivoire to complement mass media.

As a result of themes identified in the rumor tracker highlighting the widespread skepticism about the existence of COVID-19 and low-risk perception, specific messages were added to emphasize that COVID-19 exists and is a threat to everyone.

The project did not have the resources to conduct a formal evaluation of the social and behavior change activities, which were either designed or adapted because of the rumor-tracking mechanism. However, through a process evaluation, the team collected feedback from 15 stakeholders (10 CCs, 3 RCTWG members, and 2 teleoperators). Feedback was positive, with CCs emphasizing the ease of use and the sense that they were contributing to an important endeavor. Likewise, teleoperators felt the system was easy to use and relevant to the public health response but felt constrained in their ability to keep up with rumor volume due to competing demands and limited computer access. The RCTWG members appreciated the insights generated by the system and felt they were able to hear from communities rather than employing a 1-way communication response. However, they requested greater involvement in the rumor collection and analysis process. The main suggestion was to expand the system to include more public health issues, beyond COVID-19 and even beyond zoonotic diseases. The RCTWG has asked the project to scale up and continue supporting the rumor tracker. CCs also requested more regular feedback so they could grow in their capacity to contribute rumors and to keep them informed regularly of emerging credible information so they could serve as an information resource to their communities.

## DISCUSSION

Through a 6-month pilot, the real-time rumor-tracking system developed in Côte d'Ivoire identified 7 overarching themes and 20 associated belief statements informed by more than 1,700 rumor submissions. These beliefs were consistent with misinformation identified in other settings related to COVID-19, such as perceptions about the efficacy of home or herbal remedies, sun exposure, or alcohol for curing COVID-19.^19^ Rumors submitted to the system suggest that communities in Côte d'Ivoire were hesitant to accept the existence of the virus, underestimated their own risk for contracting it, and were suspicious about public health recommendations including masks and testing. While consistent and clear messaging is the best way to undermine misinformation, tracking emerging rumors can help communication actors to nuance and prioritize messages. For example, low uptake of masking can be met with traditional risk communication messaging. But specific insights emerged through the rumor tracker that provided insights about barriers in the community: a widespread conspiracy theory that masks made in China are contaminated with the virus and the perception that lack of public compliance with masking by government officials means that masks do not work. Consistent and clear messaging around masks must consider these fears, not necessarily by “myth busting” and thereby amplifying the rumors but by speaking to the real concerns of the population and modeling the appropriate behaviors. The vaccine roll-out in Côte d'Ivoire provides a new opportunity to hear nuanced feedback in real-time from communities on perceptions and fears around vaccines.

While consistent and clear messaging is the best way to undermine misinformation, tracking emerging rumors can help communication actors to nuance and prioritize messages.

The variety of conspiracy theories tracked during the pilot revealed that at least within certain communities, trust in the government, as well as the international public health response, is lacking. In addition to the cross-cutting beliefs that mirror COVID-19 misinformation in other settings, the rumor-tracking system was able to identify very local beliefs, such as an emergent rumor in a specific district related to early morning washing to prevent infection.

This pilot can serve as a proof of concept that real-time rumor tracking is both feasible and informative to the public health response. The system was built on existing infrastructure: a national hotline, the RCTWG, and locally embedded and trusted community members who, with a limited amount of training, were able to recognize reportable rumors and were motivated to contribute. Both CCs and teleoperators provided positive feedback on the ease of use and relevance of the rumor-tracking approach. While CCs could introduce bias by serving as “gatekeepers” for community feedback, there was a substantial benefit in engaging people with deep contextual knowledge of their communities, thus allowing the system to filter out the “noise” associated with typical rumor-tracking approaches like social media listening.

In a field with increasingly complex and expensive options for analyzing rumors and misinformation, this pilot prioritized system usability and the feasibility of transferring capacity and ownership to the RCTWG in its approach. Routinizing the data entry and coding process to a rapid, daily task allowed the hotline and CCP staff to efficiently process submitted rumors and accelerated the identification of problematic misinformation. The technology that supported the overall approach is a well-known open-source software platform used by more than 70 countries to track health and social data.^20^ The rumor-tracking technology was configured rapidly, and the primary cost was hosting the DHIS2 installation using Amazon Web Services. Affordable, sustainable, and secure, DHIS2 is well in alignment with the Principles for Digital Development.^21^ We have made a guidance document and DHIS2 rumor-tracking metadata package available for countries wanting to rapidly install and use this system (Supplement).

Implementers need to identify the best sources of rumors in the local context, taking into consideration national hotlines, existing feedback systems, social media penetration, and the network of trusted community actors or implementing partners. However, by using existing community contributors, institutions, and an open-source, cloud-based platform like DHIS2, the real-time rumor-tracking approach is eminently scalable. The continued interest from the Government of Côte d'Ivoire and the invitation to scale up and continue supporting the rumor-tracking system demonstrates the utility of the system. COVID-19 vaccine-related rumors comprise, at present, the bulk of submissions to the system. The government hopes that the system will contribute insights into vaccine hesitancy and help risk communicators adapt their approaches for the recently initiated vaccine rollout effort.

By using existing community contributors, institutions, and an open-source, cloud-based platform like DHIS2, the real-time rumor-tracking approach is eminently scalable.

### Limitations

The real-time rumor-tracking approach has several limitations. First, the motivation of community contributors waned during the pilot, and by the end, most rumors were submitted by a few contributors. Implementers must consider how to maintain motivation through refresher training, prompts, and airtime credit, to ensure sustained commitment to community listening. Second, while we were aware of the demographic information of the community contributors themselves, we did not ask for demographic information about those from whom they heard the rumors. Thus, we do not have a sociodemographic profile or additional information about the original source of rumor transmission. This limitation is necessary to respect the primary goal of the rumor tracker and the associated ethical constraints; the purpose of the real-time rumor-tracking system is to listen to communities and provide insights into general beliefs, not to create a surveillance system for implementers to follow up on individuals propagating rumors. Third, by organizing rumors into tables and graphs on dashboards, the temptation exists to interpret the information quantitatively. In all materials and dashboards during implementation, we include a caution that data visualized through the system should be considered a snapshot of circulating beliefs and are not representative at the population level. Finally, we were unable to explore the public health impact of tracking rumors within the scope of this pilot. Future studies should endeavor to understand whether risk communication and community engagement activities informed by rumor tracking result in greater behavior change in desired directions.

## CONCLUSION

In partnership with the RCTWG, the team is currently applying findings from this pilot by: (1) expanding the rumor-tracking scope to incorporate social media, (2) training teleoperators at 3 additional health-related hotlines to enter and code rumors, (3) recruiting local radio hosts as CCs, and (4) systematically documenting actions taken that are informed by rumor data. Real-time rumor tracking is feasible and informative and can be integrated into emergency preparedness efforts. As part of a suite of community listening approaches that includes social media listening and surveys, real-time rumor tracking can enable governments to understand their constituents and improve their public health response.

## Supplementary Material

21-00031-Tibbels-Supplement.pdf

## Translation

**En Français**

**Suivi des rumeurs sur le COVID-19 en temps réel à l'aide de méthodes communautaires en Côte d'Ivoire**

Message clé: Traiter les rumeurs est essentiel pour gérer et mettre fin à une urgence de santé publique. Nous avons piloté un système de gestion des rumeurs en utilisant des méthodes de collecte communautaires, un logiciel source-ouverte et un processus de codage et de visualisation rapide pour comprendre systématiquement et aider les acteurs à répondre aux désinformations du COVID-19 en Côte d'Ivoire.

**Principaux résultats**

- Les rumeurs et la désinformation nuisent à la santé publique en créant des obstacles aux pratiques de protection et en réduisant la confiance dans les intervenants sanitaires.
- Nous avons développé une approche de suivi des rumeurs en temps réel qui s'appuie sur les structures existantes (comme les lignes vertes nationales) et les liaisons locales (comme les agents de santé communautaires) pour soumettre les rumeurs et la désinformation à une base de données centrale. Les rumeurs sont rapidement codées à l'aide d'un formulaire standard et visualisées sur des tableaux de bord personnalisés.
- Pendant le pilote de 6 mois de cet outil en Côte d'Ivoire, nous avons reçu et codé 1 757 soumissions et utilisé les résultats thématiques pour informer les activités de communication des risques et d'engagement communautaire.

**Implications Clés**

- Les responsables de la santé devraient envisager d'utiliser une approche de suivi des rumeurs en temps réel lors des urgences de santé publique et peuvent s'appuyer sur le guide détaillé et le paquet de métadonnées DHIS2 fournis pour une technologie simple, open-source et centrée sur la communauté, afin de rendre les données sur les rumeurs assimilables par les intervenants.

**ABSTRACT**

La désinformation mondiale et la surcharge d'informations ont caractérisé la pandémie de coronavirus (COVID-19). Les rumeurs sont des informations non vérifiées qui se propagent en ligne ou de personne à personne, réduisant la confiance dans les autorités sanitaires et créant des obstacles aux pratiques de protection. La communication sur les risques et l'engagement communautaire peuvent accroître la transparence, renforcer la confiance et mettre fin à la propagation des rumeurs. S'appuyant sur des travaux antérieurs sur les virus Ebola et Zika, avec le soutien du programme de sécurité sanitaire mondiale pour le renforcement des systèmes, le projet Breakthrough ACTION, financé par l'Agence des États-Unis pour le développement international, a mis au point un processus et une technologie permettant de recueillir, d'analyser et de traiter systématiquement les rumeurs relatives au COVID-19 en temps réel en Côte d'Ivoire. Les rumeurs ont été soumises par des contributeurs communautaires et recueillies auprès des personnes appelant les lignes vertes nationales, puis traitées dans une base de données hébergée dans le nuage, construite sur le logiciel libre District Health Information System 2 (DHIS2). Les téléopérateurs des lignes vertes et les gestionnaires de données ont codé les rumeurs en temps quasi réel selon les théories du comportement dans DHIS2 et ont visualisé les résultats sur des tableaux de bord personnalisés. L'analyse et la réponse ont été effectuées en collaboration totale avec le gouvernement de Côte d'Ivoire et les partenaires de mise en œuvre afin de garantir une réponse rapide et coordonnée. Le système a permis de saisir à la fois des rumeurs très répandues qui correspondent à la désinformation dans d'autres contextes, telles que des soupçons sur le nombre de cas et la croyance que les masques ont été délibérément contaminés, ainsi que des croyances très localisées liées à des influenceurs spécifiques. Les résultats qualitatifs ont fourni un aperçu rapide des croyances en circulation, permettant aux communicateurs de nuancer et d'adapter les messages sur le COVID-19.

**Peer Reviewed**

**First published online:** May 27, 2021.
